# High Altitude Adaptability and Meat Quality in Tibetan Pigs: A Reference for Local Pork Processing and Genetic Improvement

**DOI:** 10.3390/ani9121080

**Published:** 2019-12-03

**Authors:** Mailin Gan, Linyuan Shen, Yuan Fan, Zhixian Guo, Bin Liu, Lei Chen, Guoqing Tang, Yanzhi Jiang, Xuewei Li, Shunhua Zhang, Lin Bai, Li Zhu

**Affiliations:** 1 College of Animal Science and Technology, Sichuan Agricultural University, Chengdu 611130, Sichuan, China; gml1660600546@163.com (M.G.); shenlinyuan0815@163.com (L.S.); guozhixian521@outlook.com (Z.G.); 13018240830@163.com (B.L.); chenlei815918@163.com (L.C.); tyq003@163.com (G.T.); jiangyz04@163.com (Y.J.); xuewei.li@sicau.edu.cn (X.L.); zhangsh1919@163.com (S.Z.); 2 Farm Animal Genetic Resources Exploration and Innovation Key Laboratory of Sichuan Province, Sichuan Agricultural University, Chengdu 611130, Sichuan, China

**Keywords:** altitude, carcass quality, meat quality, amino acid, fatty acid

## Abstract

**Simple Summary:**

The increase in altitude will bring about a complex change in a series of elements of nature, which will have a profound impact on human production and life. Studying domestic animals in the native environment is an effective way to explore the impact of high altitude on human life, and at the same time is conducive to the development of local animal husbandry. Here, we found that the hypoxic adaptation of Tibetan pigs may be related to higher levels of *VEGFA*, *HIF1* and myoglobin expression. The higher aerobic oxidative capacity of Tibetan pigs is beneficial to improve energy utilization, and the higher UFA content of Tibetan pigs is beneficial to cold resistance. In addition, Tibetan pigs have higher levels of BCAA and *Myh2* expression, which serve to relieve muscle fatigue and improve endurance. In addition, it was observed that there are obvious differences in carcass and meat quality traits of different altitudes pigs. Taken together, our findings illustrate the adaptability of Tibetan pigs to high altitude from various perspectives and compare carcass and meat quality traits of three pig breeds.

**Abstract:**

The carcass and meat quality traits of pig breeds living at three different altitudes (Yorkshire pigs, YP: 500m; Qingyu Pigs, QYP: 1500m; Tibetan pigs, TP: 2500m) were compared. It was observed that there are obvious differences in pig breeds with respect to performance parameters. Specifically, YP had the best carcass traits, showing high slaughter rates and leanest meat. Conversely, QYP had the highest back fat thickness and intramuscular fat (IMF) content. For the high-altitude breed TP, the animals exhibited low L* and high a* values. The genotypes contributing to the observed phenotypes were supported by a PCR analysis. The glycolytic genes expression (HK, PFK, PK) were highest in YP, whereas expression of genes related to adipogenesis (C/EBPα, FABP4, SCD1) were highest in QYP. As expected, genes associated with angiogenesis and hypoxia (*HIF1a*, *VEGFA*) were expressed at the highest levels in TP. The composition and proportion of amino and fatty acids in pig muscles at the three altitudes examined also varied substantially. Among the breeds, TP had the highest proportion of umami amino acids, whereas QYP had the highest proportion of sweet amino acids. However, TP also exhibited the highest proportion of essential fatty acids and the lowest proportion of n6:n3. This study explains the high-altitude adaptive evolution and the formation of meat quality differences in different altitude pigs from various angles and provides a reference for local pork food processing and genetic improvement of local pigs.

## 1. Introduction

In many countries and regions, the various cultures have a tradition of eating pork as a central part of their diet. According to Food and Agriculture Organization of the United Nations (FAO, http://www.fao.org/faostat/zh/#data/QL), global pork production and consumption have long exceeded 30% of the total meat production and consumption. The development of pig production and related industries has greatly improved the quality of life of people worldwide [1]. Pig carcass and meat quality traits are the most economically important features for breeders, food developers, and consumers. Traditionally, pig breeders have pursued high growth rates and lean meat. Consequently, these traits have resulted in less desirable pork. However, consumers have recently begun to pursue meat of higher quality and flavor [2]. Heirloom and local pig breeds provide these rich traits for the development of pork [3,4]. 

Yorkshire pigs (YP) are one of the main varieties of the modern pig industry, with high growth rates and lean meat percentage, which is representative of the advanced breeding levels. Qingyu Pigs (QYP) are typical fat-type Chinese local pig breeds that is widely distributed throughout the mountain areas around the Sichuan Basin [5]. Tibetan pigs (TP) are typical high-altitude pig breeds that live on the Qinghai-Tibet Plateau, and are important to the lives of about 11 million people residing on the plateau. The extremely high-altitude adaptability of Tibetan pigs is not observable in other pig breeds [6]. In-depth research on Tibetan pigs also helps us understand the hypoxic adaptation of plateau species [7]. 

The development of novel pig breeds through selective breeding is a long and complex process, which is often determined by both the local environment and people’s eating habits [8]. In-depth studies of pork quality not only provide a reference for improved breeding and food development of pigs, but can also provide insights into the local history and social culture. Protecting local germplasm resources is of great significance for the maintenance and promotion local food culture.

In the current study, the carcass traits, meat quality, amino acid, and fatty acid composition of pig breeds were examined. Additionally, the expression of genes involved in muscle development, fat deposition, and glycolysis were examined as well. All of these traits were examined in the context of the environmental and social factors that influence pork quality differences, and sought to understand food development strategies based on meat quality indicators. The results of this research can serve as a reference for local specialty food development and local pig genetic improvement.

## 2. Materials and Methods 

All experimental protocols and procedures conducted are in accordance with the requirements of the Sichuan Agricultural University Ethics Committee.

### 2.1. Animals and Treatments

**Figure 1:**
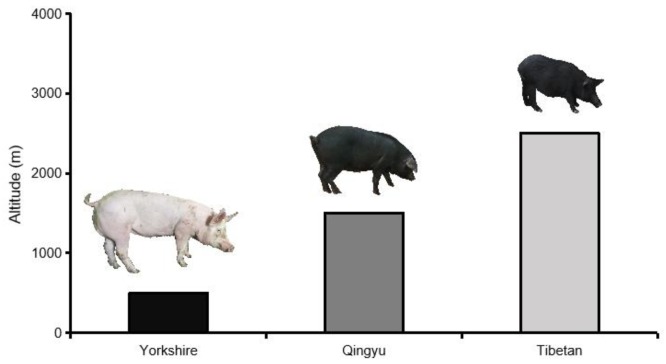
Altitude distribution of three pig breeds.

A total of 80 pigs were used in this study: 39 Yorkshire pigs (YP), including 20 males and 19 females, slaughtered at 180 days of age; 17 Qingyu pigs (QYP), including 8 males and 9 females (QYP1—5 pigs slaughtered at 180 days of age; QYP2—12 pigs, slaughtered at 300 days of age); 24 Tibetan pigs (TP), including 12 males and 12 females, slaughtered at 300 days of age. Altitude distribution of three pig breeds was shown in Figure 1. All male pigs were castrated. Ingredients of the basal experiment diets is shown in Table S1. After fasting with ad libitum access to water for 24 h, pigs were electrically stunned and exsanguinated. Approximately 10 g of a tissue core was collected from the last rib of the longissimus dorsi muscle, immediately placed in liquid nitrogen, and transferred to -80 °C for subsequent amino and fatty acid analysis, and qRT-PCR. 

### 2.2. Measurement of Carcass Characteristics

Carcass weight was recorded after evisceration. Dressing percentage was calculated from the individual live weight and carcass weight measures (pig’s head, internal organs, hooves and tail were removed from the carcasses) [4]. Accurately strip the muscles and bones of the pig carcass (the difference between the sum of each part and the weight before splitting was less than 1%). The carcass lean, fat, and bone percentages were calculated. Back fat depth (average of the seventh and last ribs, and the thickest part of the shoulder) were also assessed. Marbling score was performed using the standard 5 scoring scale system [4].

### 2.3. Measurement of Physical Correlates of Meat Quality

Muscle pH values of the longissimus dorsi muscle were measured at 45 min and 24 h postmortem using a portable pH meter (model 720A; Orion Research Inc., Boston, MA, USA). Meat color, including lightness (L*), redness (a*), and yellowness (b*) were assessed at 45 min and 24 h postmortem on the longissimus dorsi muscle using a Minolta CR-300 colorimeter (Minolta Camera, Osaka, Japan). Warner–Bratzler shear force (WBS) was determined using a texture analyzer (TA.XT. Plus, Stable Micro Systems, Godalming, UK) equipped with a Warner–Bratzler shearing device. After being stored at 4 °C for 72 h, a cuboid muscle of longissimus dorsi muscle was cooked in a circulating water bath held at 80 °C until the core temperature reached 70 °C, then cooled to room temperature. Hot dog shearing procedure was used in the test. Drip loss, shear force, and cooking loss of the longissimus dorsi muscle samples were measured as previously described [9,10]. 

### 2.4. Muscle Chemical Composition

Muscle chemical composition determination was performed as previously defined [10,11]. The crude protein was determined using the Kjeldahl method, and intramuscular fat content was determined by the Soxhlet extraction. Ash refers to the residue remaining after incineration of all organic materials in a high temperature furnace at 600 °C. 

### 2.5. Quantitative Real-Time PCR 

The frozen longissimus dorsi muscle was ground to a powder using liquid nitrogen, and total RNA was extracted from 40 mg of tissue homogenate using the triazole reagent (TaKaRa, Dalian Plateau, China). First strand cDNA was synthesized using the PrimeScript First Strand cDNA Synthesis Kit (TaKaRa). Quantitative real-time PCR (qRT-PCR) was performed using the SYBR Premix Ex Taq kit (TaKaRa) on a CFX96 real-time PCR detection system (Bio-Rad, Richmond, CA). To calculate relative mRNA expression, the 2−ΔΔCt method [12] was used with β-actin as the internal reference. The primer sequences used for qRT-PCR are listed in Table S2. 

### 2.6. Analysis of Free Amino Acids (FAA) and Fatty Acid

Prior to FAA analysis, 80 mg of tissue was homogenized, and 1000 ul pre-treatment solution (acetonitrile: water 1:1) was added. The suspensions were then shaken for 60 min. Next, the samples were centrifuged at 13200 rpm/min for 10 min, and the supernatants were collected. The supernatant fluids were used to determine FAA composition using liquid chromatography-mass spectrometry (Liquid phase: LC-20AD, Shimadzu, Japan; Mass Spectrometry: 5500 Q TRAP LC-MS/MS, AB SCIEX, USA). Pretreatment of fatty acid samples was accomplished by homogenizing 100 mg of tissue. Next, 2 ml of n-hexane was added, and shaken at 50 °C for 30 min, at which point 3 ml of KOH methanol solution (0.4 mol/L) was added. The samples were then shaken for 30 min at 50 °C. Next, 1 ml of water and 2 ml of n-hexane were added, and the samples mixed. The mixture was then allowed to stand for stratification. The upper layer was collected, and fatty acids were detected using gas chromatography-mass spectrometry (GC-MS 7890B-5977A, Agilent, USA).

### 2.7. Statistical Analysis

All data were reported as mean ± standard deviation (SD). The differences between two groups were analyzed by Student’s *t*-test. Groups of three or more were analyzed by one-way ANOVA. Statistical analyses were conducted using SPSS 20.0 software (IBM, Almond, NY, USA). Differences were considered significant when *p* < 0.05. 

## 3. Results

### 3.1. Carcass Traits

The carcass traits of the three pig breeds all showed significant differences (Table 1). The lean carcass percentage and loin eye area of YP were significantly higher (P < 0.05) than both QYP and TP. Furthermore, the carcass fat percentage, back fat thickness, and marbling scores of YP were significantly lower (*p* < 0.05) than was observed in the other breeds. The carcass weight and length of TP were significantly lower (*p* < 0.05) than both QYP and YP. The designations of QYP1 and QYP2 refer to Qingyu pigs of 180 days old and 300 days old, respectively. The carcass fat percentage, backfat thickness, and marbling scores of QYP1 and QYP2 were all significantly higher (*p* < 0.05) than YP and TP. Notably, the body weight, carcass length and backfat thickness of QYP2 were all significantly higher (*p* < 0.05) than QYP1. However, none of the other parameters assessed exhibited any significant differences between the two ages.

### 3.2. Meat Quality and Muscle Chemical Composition

The characteristics affecting meat quality are presented in Table 2. Among the three pig breeds, the L * 45 min value, b*45 min value, L*24 h value, b*24 h value, and drip loss of the YP were significantly higher (*p* < 0.05) than those of QYP and TP. However, the pH 45 min value, pH 24 h value and a* 45 min value were significantly lower (*p* < 0.05) than the QYP and TP breeds. The L* 45 min and a* 45 min values of TP were significantly lower (*p* < 0.05) than QYP and YP. The b* 45 min value and ash content of the two stages of QYP were significantly lower (P < 0.05) than YP and TP.

### 3.3. Expression of Genes in Longissimus Dorsi Muscle

**Figure 2:**
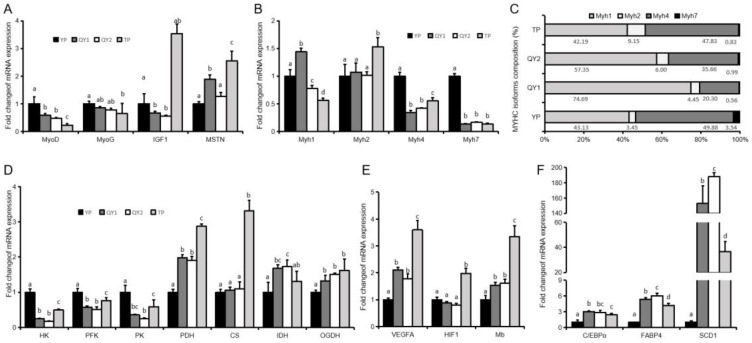
Gene expression in longissimus dorsi muscle. (**A**) The expression of genes involved in muscle development in longissimus dorsi muscle. Myogenic differentiation 1 (*MyoD*), myogenin (*MyoG*), Myocyte enhancer factor 2c (*Mef2c*), Myostatin (*MSTN*). (**B**,**C**) The expression and abundance of different subtypes of Myosin heavy chain (*MyHC*). (**D**) The expression of genes involved in muscle glycolysis and aerobic oxidation. Hexokinase (*HK*), phosphofructokinase (*PFK*), pyruvate kinase (*PK*), pyruvate dehydrogenase (*PDH*), citrate synthase (*CS*), isocitrate dehydrogenase (*IDH*), oxoglutarate dehydrogenase (*OGDH*). (**E**) The expression of vascular endothelial growth factor-A (*VEGFA*), hypoxia inducible factor-1 (*HIF1*), and myoglobin (*Mb*). (**F**) The expression of CCAAT/enhancer binding protein (*C/EBPα*), adipocyte fattyacid-binding protein (*FABP4*), and stearyl coenzyme A dehydrogenase-1 (*SCD1*), *n* = 3–6. All results are presented as means ± SEM. Different letters indicate significant difference (*p* < 0.05).

In order to better understand the differences in the meat quality of the three pig breeds, the expression of genes associated with pig carcass traits, meat quality, and high altitude adaptation were analyzed. The expression levels of *MyoD* and *MyoG* in YP were significantly higher (*p* < 0.05) than those of QYP and TP, while the expression of *MSTN* in TP was significantly higher (*p* < 0.05) than that of QYP and YP (Figure 2A). The expression levels and content ratios of *Myh4* and *Myh7* in the longissimus dorsi muscle of YP were significantly higher (*p* < 0.05) than those of the other two breeds, and the expression and proportion of *MYh2* in TP were significantly higher (*p* < 0.05) than those in YP and QYP (Figure 2B,C). The expression of *HK*, *PFK*, and *PK* in TP were significantly higher (P < 0.05) than QYP and TP (Figure 2D). The expression of *VEGFA*, *HIF1*, and Mb in TP were significantly higher (P < 0.05) than was observed in QYP and YP (Figure 2E). The expression of *C/EBPα*, *FABP4*, and *SCD1* in YP were significantly lower than QYP and TP (Figure 2F). 

### 3.4. Free Amino Acid Content

In view of small differences between meat quality traits and genes expression between QYP1 and QYP2 and the differences among other pig breeds, Qingyu pigs were represented in the follow-up study by QYP2 (pigs with usual slaughter age). As can be seen in Table 3, with the exception of Phe, Asn, Gly, and Asp, the amino acids in the longissimus dorsi muscle of the three breeds examined were significantly different (*p* < 0.05). The Lys and His content of YP was observed to be significantly higher (*p* < 0.05) than in QYP and TP. Furthermore, Gln of QYP was significantly higher (*p* < 0.05) than YP and TP, whereas ILe, Leu, Val, Trp, Met, Ser, Tyr, and Glu in TP were significantly higher (*p* < 0.05) than the other two breeds. 

**Figure 3:**
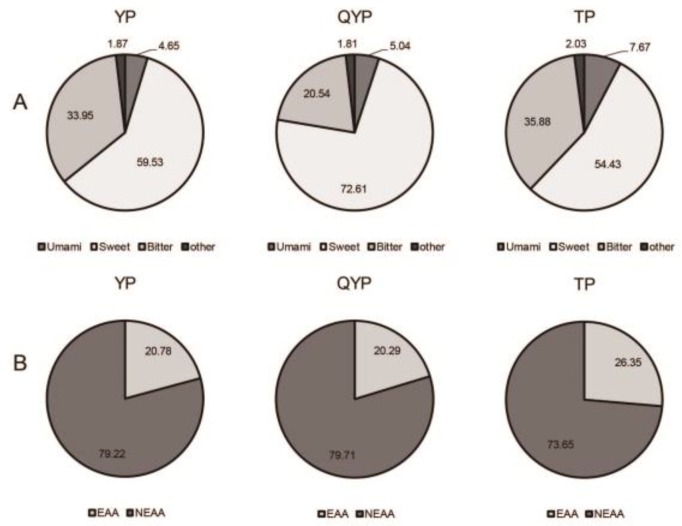
Analysis of amino acid composition in the longissimus dorsi muscle. (**A**) Amino acid ratios of longissimus dorsi muscle with different flavors in different pig breeds. (**B**) Proportion of essential amino acids (EAA) and non-essential amino acids (NEAA) in longissimus dorsi muscle of different pig breeds. All results are presented as means ± SEM. *n* = 6. Umami AA: Glu, Asp; Sweet AA: Gly, Ala, Ser, Thr, Pro, Gln, Lys; Bitter AA: Tyr, Arg, His, Val, Met, Ile, Leu, Trp, Phe.

Further analysis indicated that the proportion of umami amino acids in TP was 7.67%, which was significantly higher (*p* < 0.05) than was observed in both YP and QYP (Figure 3A). The sweet amino acid concentration of QYP was as high as 72.61%, and 77.01% for essential amino acids (EAA), which were significantly higher (*p* < 0.05) than YP and TP (Figure 3A,B).

### 3.5. Fatty Acid Levels 

In the three pig breeds examined, a total of 22 fatty acids were measured. Only C10:0 and C14:0 were observed at similar levels among all three breeds. Among the 20 fatty acids, YP and TP contained the highest proportions of C18:1n9, reaching 49.80% and 54.81%, respectively. The QYP contained the highest proportion of C16:0, reaching 22.90%. In addition, C15:0, C16:0, C17:0, C17:1, C18:2n6, C18:3n3, C20:4n6, C20:5n3, C22:0, and C24:0 in QYP pigs were observed to be significantly higher (P < 0.05) than YP and TP. Conversely, TP exhibited the highest levels of C16:1, C18:1n9, C20:0, C20:1, C20:3n3, and C22:1n9 (*p* < 0.05) (Table 4).

**Figure 4:**
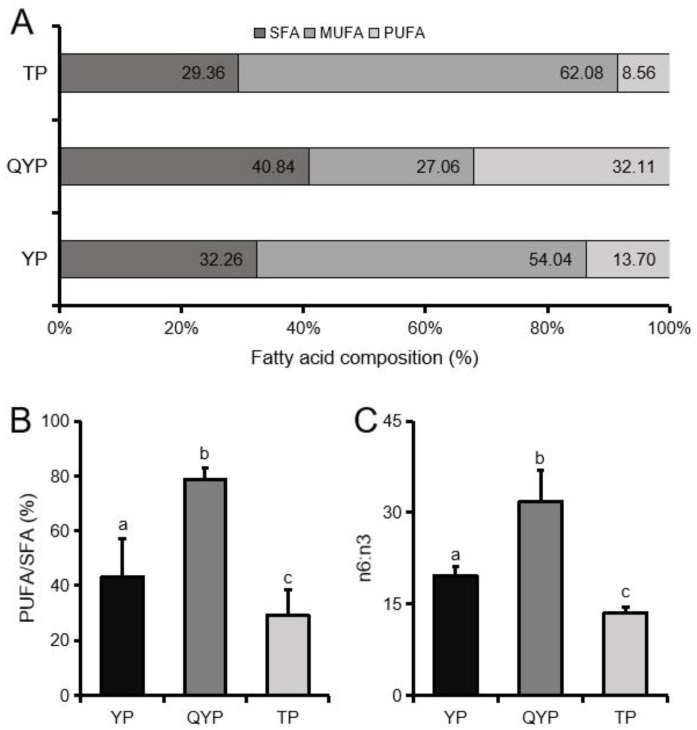
Analysis of fatty acid composition and characteristics in the longissimus dorsi muscle. (A) Composition of saturated fatty acids (SFA), monounsaturated fatty acids (MUFA), and polyunsaturated fatty acids (PUFA) in longissimus dorsi muscle of different pig breeds. (B) Percentage of PUFA/SFA in longissimus dorsi muscle of different pig breeds. (C) Ratio of n6:n3 of longissimus dorsi muscle in different pig breeds. All results are presented as means ± SEM. *n* = 6. Different letters indicate significant difference (*p* < 0.05).

Next, analysis of the ratios of polyunsaturated fatty acid (PUFA) and PUFA/SFA (saturated fatty acid) indicated significantly higher levels in QYP than in both YP and TP (*p* < 0.05). In contrast, TP were observed to have the highest levels of monounsaturated fatty acids (MUFA) of TP (*p* < 0.05). It is worth noting that the n6/n3 ratio of QYP was observed to be significantly higher (*p* < 0.05) than that of YP and TP (Figure 4).

## 4. Discussion

The differences in body composition among the different breeds were largely expected due to the breed itself and the different rearing conditions. The three pig breeds from low, intermediate, and high altitudes interestingly exhibited not only different body types, but different breeding intensities as well [13]. A workhorse of the pork industry, YP undergoes high-intensity modern breeding, whereas QYP is subjected to medium-intensity traditional breeding, and TP is bred at a low-intensity and is raised in a semi-wild state. The processing of pork for three pig breeds examined here differ substantially. For example, YP is more suitable for the production of bacon, whereas QYP is considered to be a general-purpose meat pig, and TP is predominantly made into jerky. Correspondingly, the three pig breeds correspond to vastly different human social cultures. QYP is adapted to traditional farming culture, and TP are suitable for pasturing [14]. Therefore, the selected breeds are representative of the extremes and intermediate methods and social/environmental climates in which Chinese pigs are produced.

### 4.1. Plateau Adaptability of Tibetan Pigs

The Tibetan plateau is a harsh, hypoxic environment. It has been reported that *HIF1* is a core regulator of hypoxia-responsive genes involved in the repair of cells in low-oxygen environments [15]. Vascular endothelial growth factor is a key factor in neovascularization, the expression of which is regulated by *HIF1* [16]. Previous studies have found that the heart, liver and kidney of TP all expressed higher levels of *VEGFA* than YP [17]. In the present study, a similar pattern of *VEGFA* and *HIF1* expression was observed in the longissimus dorsi muscle. In addition, the muscle redness of Tibetan pigs was the highest among the pig breeds examined, and the muscle L* value was the lowest. Approximately 80–90% of muscle color is attributable to myoglobin (*Mb*) [18]. This protein has a higher affinity for oxygen than hemoglobin, and was present in the highest concentrations in TP, which would explain the enhanced red coloration of TP meat. In summary, the Tibetan pig muscle exhibited the lowest L* value and the highest a* value, compared with YP and QYP.

Another feature of the plateau environment is the scarcity of food. As a result, it is crucial that Tibetan pigs are more efficient in their energy usage and have exceptional endurance. In order to adapt to different environments, the composition and function of muscle tissue will adjust accordingly. Based on myosin composition, the skeletal muscle fibers can be divided into Myh1, Myh2, Myh4, and Myh7 [19]. Muscles consisting predominantly of Myh2 are classified as fast oxidized muscle fibers, which is in between the oxidized muscle fiber (Myh7) and the glycolytic muscle fiber (Myh4). Therefore, Myh2 can provide energy to the body under both aerobic and anaerobic conditions [18]. Several previously published studies have demonstrated that in order to adapt to the special physiology of excavation activities under hypoxic conditions, the skeletal muscle composition of mole rats has undergone compensatory changes. The cervical trapezius, gastrocnemius, and quadriceps muscles are mainly myosin heavy chain 2a (Myh2) muscle fibers [20]. Similarly, it was observed here that the longissimus dorsi muscle of TP contained significantly higher levels of *Myh2* than YP and QYP. 

Glycolysis occurs during the first stage of aerobic oxidation of carbohydrates to provide a rapid energy supply. However, the efficiency of this process is low [11,21]. The expression of glycolytic-related genes in the muscle of Tibetan pigs was at an intermediate level between YP and QYP. The expression of *CS* and *OGDH*, which are located downstream of the aerobic oxidation of carbohydrates were higher than was observed in both YP and QYP. This is logical, as it provides a quick supply energy to respond to dangerous natural environments. 

Branches chain amino acids, or BCAAs (leucine, isoleucine, and valine), are essential amino acids [22]. Not only do they serve as raw materials for protein synthesis, but they also promote growth through the promotion of insulin and growth hormone secretion, and enhance metabolism [23]. These three amino acids can slow muscle fatigue, speed up recovery, reduce muscle protein catabolism during exercise, and help the body absorb protein, so they are often used as a muscle protectant [24]. Interestingly, they were observed at significantly higher levels in TP muscle than YP and QYP. Tryptophan has the effect of promoting differentiation of bone marrow T lymphocyte precursors into mature T lymphocytes [25,26]. Therefore, a lack of tryptophan can lead to a decrease in cell mediated immune responses. Here, it was observed that muscle tryptophan of was significantly higher in TP than both YP and QYP. 

A cold environment is also a survival challenge for Tibetan pigs. It was observed that this breed has higher levels of unsaturated fatty acids (UFAs). Studies have shown that UFAs are the main factors affecting the fluidity of cell membranes, and are involved in resistance to cold damage in animals [27], plants [28], and microorganisms [29]. As a free-range grazing breed, Tibetan pigs have the opportunity to eat more wild plants, which have higher concentrations of UFAs [30]. High levels of UFAs are likely part of the cold resistance mechanism for Tibetan pigs. 

### 4.2. Carcass Traits and Meat Quality Differences between Yorkshire, Penzhou Mountain, and Tibetan Pigs

Carcass traits are important indicators in the evaluation of meat performance in pigs. Carcass weight, dressing percentage, and carcass lean percentage are the main indicators of concern [31]. Here, the dressing percentage, carcass lean rate, and loin eye area of YP are much higher than those of QYP and TP. It has been reported that *MyoD*, *MyoG*, and *Mef2c* can promote myogenic differentiation, and play a positive role in the development of porcine skeletal muscle [32,33]. The expression of *MyoD* and *MyoG* in YP are significantly higher than was observed in QYP and TP. Myostatin (MSTN) is a negative regulator of skeletal muscle growth [34], which was found to be the highest in Tibetan pig muscles in the present study. The longissimus dorsi muscle fiber type differs between wild and commercial pig breeds [35]. It has been reported that *Myh4* is associated with lean muscle mass and growth rate [36], and that the proportion of *Myh4* expression in the longissimus dorsi muscle of YP was the highest. These results suggest that QYP and TP have great potential for genetic improvement in carcass traits. In addition, Tibetan pigs are small in size, which make them an attractive animal model system for the study of human diseases [37].

Pork pH is closely related to the products of glycolysis in muscle tissue [9,21,38]. The medium level of muscle glycolysis allows the pH of Tibetan pigs to average between that of YP and QYP, while the relatively high levels of tricarboxylic acid cycle-related genes are expressed. Meat color is an important sensory indicator for the evaluation of pork [39,40]. Pork from TP and QYP have higher a* and lower b* values, which implies that Chinese pork sales are based on cultural and visual perceptions of meat freshness. Local pigs show better meat color (higher a* value) and pH value, and further utilize the meat processing technology such as chilled meat processing to better demonstrate the meat quality potential of local pigs.

The muscle amino acid composition of the three pig breeds varied significantly. In the context of the flavors they impart, amino acids are classified into umami amino acids, sweet amino acids, and bitter amino acids [41,42]. Among the breeds examined here, TP had the highest umami amino acid content, and QYP had the highest sweet amino acid content. These may be the reason why QYP and TP meat quality are preferred in many aspects over YP. Furthermore, TP has higher levels of EAAs. Because of the harsh environment and remote location, modern dietary supplements, and ready access to a variety of foods on the Tibetan plateau can be scarce. Therefore, Tibetan pork is an effective way to supplement EAAs in the plateau. The high content of BCAA in Tibetan pork is not only conducive to the survival of Tibetan pigs, but also to the health of people who feed on them [43]. In addition, the high levels of bitter amino acids in Tibetan pork make cooking and seasoning of the meat, such as barbecue, a preferred method of processing and preparation. Although, with the development of food science and technology, amino acid enzymatic technology can expand the choices available for the processing of pork [44]. However, considering local food culture and characteristics, further research on pork jerky may be more conducive to the development of the plateau economy.

Traditional Chinese dishes are full of color, fragrance, and taste, of which local pork has been a preferred protein source in traditional Chinese dishes. Most Chinese local pigs are high-fat breeds. Due to their strong fat deposition capacity and higher saturated fatty acids (SFAs), a diet high in these meats has been demonstrated to affect cholesterol metabolism [45]. Despite the high SFA content of QYP pork, the local climate is warm with abundant green vegetables for consumption throughout the year, which can enhance the health of the people. In contrast, the people of the plateau have a single source of food, with a healthier ratio of PUFA/SFA [46]. Polyunsaturated fatty acids possess many physiological functions, such as maintaining biofilm structures, treating cardiovascular diseases, anti-inflammation, and promotion of brain development [47]. This reflects the harmonious evolution of human and pigs. The content of intramuscular fat (IMF) is closely related to meat tenderness, pH value, marbling, muscle flavor, and other meat quality traits [48,49]. Of the breeds examined here, QYP has the highest IMF as well as *C/EBPα* [50], *FABP4* [51], and *SCD1* [52] expression. All of these genes are related to fat synthesis and were highly expressed in QYP relative to the other breeds examined here. It is worth noting that *SCD1* is a key regulatory gene that catalyzes the synthesis of PUFA [53]. The data presented here suggests that QYP pork is richer and more comprehensive in fatty acids, while the fatty acid composition of TP pork is more favorable to human health.

## 5. Conclusions

A comparative analysis of three pig breeds showed that the plateau adaptability of Tibetan pigs is likely related to genetics, environment, and production methods. The hypoxic adaptation of Tibetan pigs may be related to higher levels of *VEGFA*, *HIF1*, and myoglobin expression. The higher aerobic oxidative capacity of Tibetan pigs is beneficial to improve energy utilization, and the higher UFA content of Tibetan pigs is beneficial to cold resistance. In addition, Tibetan pigs have higher levels of BCAA and *Myh2* expression, which serve to relieve muscle fatigue and improve endurance. Tibetan pigs have undergone numerous evolutionary changes to enhance their survivability in the harsh environment. Not only have these changes enhanced the survival of Tibetan pigs, but they have also altered meat and carcass quality traits, as well as the health of local people who eat their meat. Further analysis of carcass traits and meat quality indicators showed that YP had excellent carcass traits (higher dressing percentage, carcass lean, and loin eye area), while QYP and TP had better meat quality (higher PH, a* value and intramuscular fat). From the human health and food production perspectives, QYP pork has richer and more comprehensive fatty acid content. In contrast, TP pork exhibits contains higher levels of EAAs and a more favorable fatty acid composition for health. However, it should be noted that excessive fat intake is harmful to human health. There is great potential for cross breeding and further food development of three pig breeds examined here.

## Figures and Tables

**Table 1:** Pig carcass traits.

Carcass traits	YP (*n* = 39)	QYP1 (*n* = 5)	QYP2 (*n* = 12)	TP (*n* = 24)	Significance
Carcass weight, kg	84.60 ± 9.39 ^a^	44.64 ± 3.99 ^b^	70.32 ± 16.96 ^a^	28.88 ± 4.04 ^c^	<0.001
Carcass length, cm	81.09 ± 2.27 ^a^	64.4 ± 2.97 ^b^	73.83 ± 11.74 ^a^	57.4 ± 2.71 ^c^	<0.001
Dressing, %	74.87 ± 3.05 ^a^	66.85 ± 5.03 ^b^	71.61 ± 7.8 ^ab^	69.69 ± 2.23 ^b^	0.002
Bone rate, %	13.1 ± 2.07 ^a^	9.26 ± 1.78 ^b^	11.41 ± 3.57 ^ab^	10.84 ± 1.62 ^b^	0.037
Carcass lean, %	64.11 ± 5.59 ^a^	43.32 ± 2.94 ^b^c	40.68 ± 5.12 ^b^	47.87 ± 3.63 ^c^	<0.001
Carcass fat, %	22.79 ± 4.80 ^a^	47.34 ± 3.96 ^b^	47.79 ± 8.41 ^b^	41.16 ± 3.64 ^c^	<0.001
Back fat thickness, cm	1.89 ± 0.48 ^a^	3.33 ± 0.54 ^b^	4.14 ± 1.14 ^c^	2.59 ± 0.5^d^	<0.001
Loin eye area, cm2	49.39 ± 8.27 ^a^	17.84 ± 3.67 ^b^c	20.85 ± 3.42 ^c^	14.08 ± 3.48 ^b^	<0.001
Marbling scores	1.05 ± 0.5 ^a^	2.63 ± 0.48 ^b^	3.13 ± 0.53 ^bc^	2.46 ± 0.45 ^b^	<0.001

Different letters indicate significant difference (*p* < 0.05).

**Table 2:** Meat quality and chemical composition of the longissimus dorsi muscle.

Meat quality	YP (*n* = 32)	QYP1 (*n* = 5)	QYP2 (*n* = 12)	TP (*n* = 12)	Significance
PH45min	6.28 ± 0.17 ^a^	6.8 ± 0.31 ^b^	6.6 ± 0.3 ^bc^	6.51 ± 0.15 ^c^	<0.001
PH24h	5.7 ± 0.29 ^a^	6.11 ± 0.17 ^b^	5.92 ± 0.33 ^b^	6 ± 0.11 ^b^	0.002
L*45min	46.99 ± 1.98 ^a^	43.65 ± 3.25 ^b^	41.03 ± 2.76 ^b^	38.84 ± 2.7 ^c^	<0.001
a*45min	5.93 ± 1.15 ^a^	8.42 ± 2.08 ^b^	10.59 ± 2.99 ^b^	12.57 ± 2.27 ^c^	<0.001
b*45min	6.75 ± 0.73 ^a^	0.6 ± 0.42 ^b^	1.7 ± 1.57 ^c^	3.14 ± 0.64^d^	<0.001
L*24h	52.63 ± 3.31 ^a^	47.71 ± 4.61 ^b^	45.77 ± 2.46 ^b^	44.89 ± 4 ^b^	<0.001
a*24	9.82 ± 1.94 ^a^	8.83 ± 1.97 ^a^	11.05 ± 2.96 ^ab^	12.14 ± 2.73 ^b^	0.026
b*24h	7.83 ± 0.91 ^a^	3.49 ± 1.21 ^b^	4.97 ± 2.03 ^b^	5.11 ± 2.57 ^b^	<0.001
Drip loss, %	3.75 ± 1.81 ^a^	1.91 ± 0.48 ^b^	2.12 ± 0.59 ^b^	2.37 ± 0.35 ^b^	0.013
Cooking loss, %	34.40 ± 1.82	33.96 ± 2.29	36.94 ± 8.23	33.26 ± 4.08	0.341
Shear force, kg	9.9 ± 3.49	6.05 ± 2.68	6.44 ± 2	4.7 ± 1.86	0.694
Crude protein, %	21.58 ± 3.77 ^ab^	24.22 ± 0.52 ^a^	21.89 ± 0.96 ^ab^	20.27 ± 2.36 ^b^	0.077
Intramuscular fat, %	1.43 ± 0.55 ^a^	2.48 ± 0.3 ^b^	4.62 ± 1.85 ^c^	1.88 ± 0.33 ^ab^	<0.001
Ash, %	2.19 ± 0.5 ^a^	1.35 ± 0.07 ^b^	1.15 ± 0.06 ^b^	2.73 ± 1.47 ^a^	<0.001

Different letters indicate significant difference (*p* < 0.05).

**Table 3:** Free amino acid concentration of longissimus dorsi muscle (%).

AA	YP (*n* = 6)	QYP (*n* = 6)	TP (*n* = 6)	*p* Value
Ile	2.09 ± 0.33 ^a^	2.16 ± 0.29 ^a^	3.69 ± 0.31 ^b^	<0.001
Leu	2.76 ± 0.23 ^a^	3.47 ± 0.48 ^b^	4.01 ± 0.52 ^c^	0.001
Lys	5.95 ± 0.5 ^a^	3.6 ± 0.53 ^b^	3.8 ± 0.48 ^b^	<0.001
Thr	2.93 ± 0.26 ^a^	3.53 ± 0.4 ^b^	3.76 ± 0.32 ^b^	0.002
Val	2.62 ± 0.32 ^a^	3.49 ± 0.36 ^b^	4.18 ± 0.39 ^c^	<0.001
Trp	0.93 ± 0.09 ^a^	0.25 ± 0.04 ^b^	1.05 ± 0.10 ^c^	<0.001
Met	1.33 ± 0.11 ^a^	0.97 ± 0.38 ^b^	3 ± 0.28 ^c^	<0.001
Phe	2.18 ± 0.44 ^a^	2.61 ± 0.52 ^ab^	2.77 ± 0.21 ^b^	0.063
Arg	4.02 ± 0.55 ^a^	2.92 ± 0.49 ^b^	3.89 ± 0.57 ^a^	0.005
His	15.31 ± 1.29 ^a^	2.69 ± 0.26 ^b^	8.36 ± 1.1 ^c^	<0.001
Asn	1.86 ± 0.17	1.8 ± 0.35	2.03 ± 0.11	0.246
Ser	2.58 ± 0.2 ^a^	3.19 ± 0.54 ^b^	3.93 ± 0.46 ^c^	<0.001
Gly	7.36 ± 1.53	7.61 ± 1.09	8.01 ± 1.56	0.727
Ala	20.22 ± 2.25 ^ab^	21.73 ± 2.11 ^a^	17.82 ± 2.5 ^b^	0.031
Tyr	2.73 ± 0.11 ^a^	1.79 ± 0.26 ^b^	4.83 ± 0.34 ^c^	<0.001
Gln	17.52 ± 2.61 ^a^	30.9 ± 4.33 ^b^	14.12 ± 0.97 ^a^	<0.001
Glu	3.96 ± 1.44 ^a^	4.21 ± 0.39 ^a^	7.44 ± 0.86 ^b^	<0.001
Asp	0.65 ± 0.15 ^ab^	0.86 ± 0.82 ^a^	0.19 ± 0.09 ^b^	0.079
Pro	3.00 ± 0.13 ^a^	2.21 ± 0.32 ^b^	3.13 ± 0.31 ^a^	<0.001

Different letters indicate significant difference (*p* < 0.05).

**Table 4:** Fatty acid composition (%) of longissimus dorsi muscle.

FAA	YP (*n* = 6)	QYP (*n* = 6)	TP (*n* = 6)	*p* Value
C10:0	0.07 ± 0.01	0.06 ± 0.05	0.06 ± 0.01	0.689
C12:0	0.07 ± 0.01 ^a^	0.01 ± 0.02 ^b^	0.05 ± 0.01 ^a^	<0.001
C14:0	1.33 ± 0.13	1.31 ± 0.18	1.34 ± 0.14	0.964
C15:0	0.03 ± 0.01 ^a^	0.10 ± 0.01 ^b^	0.03 ± 0.02 ^a^	<0.001
C16:0	13.95 ± 0.77 ^a^	22.90 ± 0.69 ^b^	15.60 ± 0.86 ^c^	<0.001
C16:1	3.13 ± 0.48 ^a^	3.40 ± 0.41 ^a^	5.60 ± 1.04 ^b^	<0.001
C17:0	0.17 ± 0.04 ^a^	0.35 ± 0.02 ^b^	0.15 ± 0.05 ^a^	<0.001
C17:1	——	0.28 ± 0.01 ^a^	0.17 ± 0.04 ^b^	<0.001
C18:0	16.37 ± 1.72 ^a^	15.81 ± 0.47 ^a^	11.77 ± 0.53 ^b^	<0.001
C18:1n9	49.80 ± 3.02 ^a^	22.79 ± 1.8 ^b^	54.81 ± 3.34 ^c^	<0.001
C18:2n6	9.91 ± 2.53 ^a^	21.46 ± 2.04 ^b^	6.48 ± 1.65 ^c^	<0.001
C18:3n3	0.56 ± 0.14 ^a^	0.60 ± 0.09 ^a^	0.33 ± 0.07 ^b^	<0.001
C20:0	0.24 ± 0.09 ^a^	0.17 ± 0.02 ^a^	0.32 ± 0.05 ^b^	0.002
C20:1	1.09 ± 0.25 ^a^	0.55 ± 0.1 ^b^	1.41 ± 0.12 ^c^	<0.001
C20:2	0.58 ± 0.12 ^a^	0.52 ± 0.08 ^a^	0.4 ± 0.07 ^b^	0.016
C20:3n3	0.06 ± 0.01 ^a^	0.06 ± 0.01 ^a^	0.24 ± 0.12 ^b^	0.001
C20:4n6	2.56 ± 1.21 ^a^	9.13 ± 1.03 ^b^	1.08 ± 0.61 ^c^	<0.001
C20:5n3	0.04 ± 0.02 ^a^	0.23 ± 0.04 ^b^	0.03 ± 0.02 ^a^	<0.001
C22:0	0.02 ± 0.01 ^a^	0.04 ± 0.00 ^b^	0.02 ± 0.00 ^a^	<0.001
C22:1n9	0.02 ± 0.01 ^a^	0.03 ± 0.00 ^a^	0.09 ± 0.02 ^b^	<0.001
C23:0	——	0.04 ± 0.01	——	——
C24:0	0.02 ± 0.01 ^a^	0.05 ± 0 ^b^	0.02 ± 0.01 ^a^	<0.001

Different letters indicate significant difference (*p* < 0.05).

## Supplementary Materials

The following are available online at https://www.mdpi.com/2076-2615/9/12/1080/s1, Table S1: Ingredients of the basal experiment diets; Table S2: The primer sequences used for qRT-PCR.
