# Supplementary material for: High Altitude Adaptability and Meat Quality in Tibetan Pigs: A Reference for Local Pork Processing and Genetic Improvement
Source: Animals (Basel). 2019 Dec 3;9(12):1080. doi: 10.3390/ani9121080 (PMC6940921; doi:10.3390/ani9121080)
Supplement: Supplementary file 1 [file animals-09-01080-s001.pdf]

High Altitude Adaptability and Meat Quality in Tibetan Pigs: A Reference for Local Pork Processing and Genetic Improvement

Mailin Gan [1,2,†], Linyuan Shen [1,2,†], Yuan Fan [1,2], Zhixian Guo [1,2], Liu bin [1,2], Lei Chen [1,2], Guoqing Tang [1,2], Yanzhi Jiang [1,2], Xuewei Li [1,2], Shunhua Zhang [1,2], Lin Bai [1,2*] and Li Zhu [1,2,*]

Table S1. Ingredients of the basal experiment diets g/kg.

Item	QYP			YP	
	**10–30kg**	**30–80kg**	**80–140kg**	**20–50kg**	**50 ~ kg**
Corn	650	700	772	700	772
Soybean	320	270	200	270	200
CaHPO4	9	9	8	9	8
$CaCO_3$	8	8	7	8	7
NaCl	3	3	3	3	3
Lysine	0.3	0.2	——	0.3	——
Premix	10	10	10	10	10

Table S2. The primer sequences used for qRT-PCR (F: forward, R: reverse)

Gene	Primer Sequence (5'–3')	TM/°C
Myh1	F-TTGACTGGGCTGCCATCAAT	60.5
	R-GCCTCAATGCGCTCCTTTTC	
Myh2	F-CTCTGAGTTCAGCAGCCATGA	60
	R-GATGTCTTGGCATCAAAGGGC	
Myh4	F-GAGGTACATCTAGTGCCCTGC	60
	R-GCAGCCTCCCCAAAAATAGC	
Myh7	F-GTTTGCCAACTATGCTGGGG	60
	R-TGTGCAGAGCTGACACAGTC	
MyoG	F-GAAAACTACCTGCCCGTCCA	58
	R-CCACAGACACGGACTTCCTC	
MyoD	F-CACGTCTAGCAACCCGAATCA	58
	R-GGCGTTGCGCAGGATTT	
IGF1	F-GAACTGAAGAGCGTCCACCA	57
	R-TGCTTGCTCTCCTTCACCAG	
MSTN	F-CCAGAGAGATGACAGCAGTGATG	61
	R-TTCCTTCCACTTGCATTAGAAGATC	
HK	F-AGATGATCGCCTCGCATCTG	60
	R-GCTCCAAGCCCTTTCTCCAT	
PFK	F-GGAGAGCTGAGACTATAAGAGTGG	61
	R-CCAGAGGTTAACACGGCGAT	
PK	F-CCTGATAGCTCGTGAGGCTG	60
	R-AGGTCTGTGGAGTGACTGGA	
PDH	F-CCTTGCAGTTGCAACCAGTC	60

	R-TCATTACAGAGACTACAGCGAGC	
CS	F-CTGACACAGCTGCAGAAGGA	58
	R-AACATGGGGTCATGAGGCAG	
IDH	F-CGGGCACGTTCAAGATTGTC	60
	R-TTGGTGCTCATGTAGAGCGG	
OGDH	F-GAAGACAGGCAGGCGTGA	58
	R-GCTAGTCCCGCTGAGAAAGG	
VEGFA	F-CTCACCAAGGCCAGCACATA	60
	R-AAATGCTTTCTCCGCTCCGA	
HIF1	F-CCACCTCTGGACGTGCTTTT	56.8
	R-CTTCCATGGCGAATCTGTGC	
Mb	F-TGAAGACAGCAGAACGTCCC	58
	R-TTAGGTTCCAGACACCACGC	
C/EBPα	F-CAAGAACAGCAACGAGTACCG	59
	R-GTCACTGGTCAACTCCAGCAC	
FABP4	F-AAGAAGTGGGAGTGGGCTTT	60.7
	R-TCCCACTTCTGCACCTGTAC	
SCD1	F-ACACTTGGGAGCCCTGTATG	60
	R-GGGCAGTCGAGCTTTGTAAG	
β-actin	F-AAGGACCTCTACGCCAACAC	60
	R-CTGGCTGATCCACATCTGCT	
